# The Moderating Role of Demographic Variables in the Effect of Health Literacy on Anti-Vaccination Sentiment

**DOI:** 10.3390/vaccines14070582

**Published:** 2026-06-30

**Authors:** Ilkay Altunsoy, Berkay Kargili, Abdulhalim Senyigit

**Affiliations:** 1 Department of Medical Services and Techniques, Vocational School, Istanbul Atlas University, 34408 Istanbul, Türkiye; 2 Department of Health Management, Faculty of Health Sciences, Istanbul Yeni Yuzyil University, 34010 Istanbul, Türkiye; 3 Department of Internal Medical Sciences, Faculty of Medicine, Istanbul Atlas University, 34408 Istanbul, Türkiye

**Keywords:** health literacy, vaccine refusal, vaccine hesitancy, moderation analysis, education, public health

## Abstract

Background/Objective: Vaccine hesitancy continues to pose a challenge to public health, and health literacy has been suggested as a potential factor influencing vaccination attitudes. However, the nature of this relationship and the role of sociodemographic characteristics remain to be clarified. This study aimed to examine the association between health literacy and vaccine refusal and to explore whether this relationship is moderated by education level, age, income, place of residence, and gender. Methods: A cross-sectional nationwide online survey was conducted among 413 adults living in Türkiye. Data was analyzed using SPSS version 22. Distributional assumptions were evaluated using the Kolmogorov–Smirnov test together with skewness and kurtosis values. Moderation analyses were performed using Hayes’ PROCESS macro (Model 1), and statistical significance was set at *p* < 0.05. Results: The study included 413 participants, most of whom were aged 18–30 years (62.7%) and female (69.3%). Health literacy showed a statistically significant negative association with vaccine refusal (β ranging from −0.30 to −0.72, *p* < 0.001). Education level was identified as a significant moderator (R^2^ = 0.14), indicating that the strength of this association varied across educational groups. Specifically, the inverse relationship appeared more pronounced in individuals with lower educational attainment and attenuated at higher education levels, becoming non-significant in the postgraduate group. In contrast, age, income, place of residence, and gender did not demonstrate consistent or statistically robust moderating effects. Although rural residence was associated with higher vaccine refusal levels, it did not significantly modify the relationship between health literacy and vaccine refusal. Conclusions: The findings suggest that higher health literacy is associated with lower vaccine refusal; however, this relationship appears to vary by educational level. The modest explanatory power of the models indicates that health literacy alone may not fully account for vaccine-related attitudes. Further research incorporating additional behavioral and contextual factors is warranted to better understand the determinants of vaccine refusal.

## 1. Introduction

Vaccine refusal and vaccine hesitancy remain major challenges for public health, despite the proven effectiveness of vaccination programs in preventing infectious diseases. The World Health Organization (WHO) has identified vaccine hesitancy as a global health threat, defining it as a delay in acceptance or refusal of vaccination despite the availability of vaccination services. This phenomenon is complex and context-specific, varying across time, place, and vaccine type, and is influenced by confidence, complacency, convenience, and broader social determinants [1,2].

Health literacy has increasingly been considered an important factor in vaccine-related decision-making. It refers to individuals’ ability to access, understand, appraise, and apply health information to make appropriate health decisions. Low health literacy has been associated with poorer health outcomes and reduced ability to understand medical information, which may also affect how individuals interpret vaccine benefits, risks, and misinformation [3].

Previous studies have reported an association between health literacy and vaccination attitudes [4]. A systematic review by Lorini et al. [5] showed that the relationship between health literacy and vaccination is not uniform and may differ according to country, population, age group, and vaccine type. Limited health literacy may impair communication about vaccination and contribute to vaccine hesitancy [6]. More recent studies have generally suggested that higher health literacy is associated with lower vaccine hesitancy or refusal, although some findings remain inconsistent across settings [7,8,9].

Social media networks have also become important determinants of vaccine-related attitudes. These platforms can facilitate vaccine uptake by rapidly disseminating public health information and increasing awareness regarding the benefits of vaccination. However, they may also contribute to vaccine hesitancy by amplifying misinformation, unverified claims, and concerns about vaccine safety and adverse events. Consequently, the quality and credibility of information shared through social media may substantially influence public perceptions of vaccines and vaccination decisions [10,11].

In addition to individual-level factors such as health literacy, institutional trust has emerged as an important determinant of vaccine-related attitudes. Institutional trust refers to public confidence in healthcare systems, governmental agencies, scientific organizations, and public health authorities responsible for communicating and implementing vaccination policies. Previous research has shown that individuals with higher levels of trust in health institutions are generally more likely to accept vaccination recommendations, whereas reduced trust is associated with greater vaccine hesitancy and refusal [10,11]. The COVID-19 pandemic further highlighted the importance of transparent, accurate, and consistent communication in maintaining public confidence, as perceived inconsistencies, misinformation, or lack of transparency may undermine institutional trust and contribute to vaccine hesitancy [12]. Therefore, strengthening both health literacy and trust in public institutions may be important for improving vaccine acceptance and addressing vaccine refusal.

The principle of informed consent is also relevant to vaccination decision-making. Informed consent requires that individuals receive clear, accurate, and balanced information regarding the potential benefits and risks of vaccination, enabling them to make autonomous and informed health decisions. In this context, health literacy plays a crucial role by facilitating the ability to access, understand, evaluate, and apply vaccine-related information. Individuals with higher health literacy may be better equipped to weigh the benefits of vaccination against potential risks, whereas limited health literacy may hinder informed decision-making and contribute to vaccine hesitancy or refusal. Therefore, promoting both informed communication and health literacy may support more informed vaccination decisions and improve public confidence in immunization programs [8,13,14].

Sociodemographic characteristics may further shape this relationship. Education level is closely related to both health literacy and vaccine-related attitudes. Individuals with higher educational attainment may have greater access to reliable health information and a stronger capacity to evaluate vaccine-related claims [15,16]. However, education may not simply act as an independent predictor; it may also modify the strength of the association between health literacy and vaccine refusal. Other factors such as age, income, gender, and place of residence have also been examined as potential determinants of vaccine hesitancy, but their effects appear to vary across populations and contexts [2,8,17,18,19].

In Türkiye, vaccine hesitancy has emerged as an increasing public health concern, influenced by concerns regarding vaccine safety, misinformation, and declining trust in health authorities. Previous studies have documented vaccine hesitancy in different Turkish populations, including pregnant women and academics. However, few studies have examined the relationship between health literacy and vaccine refusal in the general adult population, and evidence regarding the moderating role of sociodemographic factors remains limited [20,21]. Addressing these gaps may facilitate the development of targeted interventions to improve vaccine acceptance in Türkiye.

Although prior research supports a link between health literacy and vaccine hesitancy, fewer studies have examined whether this association differs across sociodemographic groups. Clarifying the moderating role of education and other demographic variables may help identify populations in which health literacy-based interventions could be most effective [8,22,23,24]. Therefore, the present study aimed to examine the association between health literacy and vaccine hesitancy and to determine whether this relationship is moderated by education level, age, income, place of residence, and gender.

## 2. Materials and Methods

### 2.1. Ethical Approval

This study was conducted in accordance with the ethical principles of the Declaration of Helsinki and received approval from the Non-Interventional Ethics Committee of Istanbul Atlas University (Date: 19 March 2025; Approval No: 03/26; E-22686390-050.99-62738). All participants were informed about the aims and procedures of the study prior to participation, and electronic informed consent was obtained via Google Forms. Participation was voluntary and anonymous.

### 2.2. Study Design and Participants

This cross-sectional study was conducted among 413 adult participants between May 2025 and October 2025. Individuals aged 18 years and older who voluntarily agreed to participate were included. Participants were recruited using a convenience sampling approach from [specify setting, e.g., outpatient clinics/community settings/online platforms]. Individuals with incomplete responses or an inability to provide informed consent were excluded. Data were collected online across Türkiye.

The target population consists of individuals aged 18 and over residing in Türkiye. A snowball sampling approach was used to ensure a balanced distribution in terms of age, gender, education level, income and place of residence. Based on widely accepted sample size estimation tables [25,26,27], a minimum sample size of 384 was determined for large populations. A total of 413 participants were included in the study.

Data was collected through an online questionnaire distributed via social media platforms (Facebook v572.0, Twitter v12.18, Instagram v395.0, LinkedIn v14.2), email groups, and online forums. Additionally, a snowball sampling method was used to increase participation.

Inclusion criteria were: (i) being 18 years or older, (ii) residing in Türkiye, and (iii) providing voluntary consent to participate. Individuals under the age of 18 or those who did not consent were excluded. No personally identifiable information was collected, and all data was analyzed anonymously.

### 2.3. Data Collection Tools

#### 2.3.1. Sociodemographic Information Form

A structured form developed by the researchers was used to collect participants’ sociodemographic characteristics, including age (18–30, 31–50, 51–64, ≥65), gender (female/male), education level (primary school, secondary school, high school, undergraduate, postgraduate), income level (low, middle, high), and place of residence (urban/rural).

#### 2.3.2. Health Literacy Scale (HLS)

Health literacy was assessed using the Health Literacy Scale developed by Türkoğlu and Kılıç [28], which has established validity and reliability in the Turkish population. The scale consists of 14 items rated on a 5-point Likert scale (1 = strongly disagree to 5 = strongly agree) (File S1).

The scale includes three subdimensions:

1. Functional health literacy (5 items)
2. Interactive health literacy (5 items)
3. Critical health literacy (4 items)

Higher scores indicate higher levels of health literacy. The Cronbach’s alpha coefficient for the scale was reported as 0.794.

#### 2.3.3. Vaccine Refusal Scale (VRS)

Vaccine-related attitudes were measured using the Vaccine Refusal Scale developed by Kılınçarslan et al. [29]. The scale consists of 21 items rated on a 5-point Likert scale (1 = strongly disagree to 5 = strongly agree).

The scale comprises the following subdimensions:

1. Perceived benefits and protective value of vaccines
2. Vaccine refusal
3. Alternatives to vaccination
4. Justification of vaccine hesitancy

Reverse-coded items were recorded prior to analysis. Higher scores indicate higher levels of vaccine refusal. The Cronbach’s alpha coefficient for the scale was 0.938.

### 2.4. Statistical Analysis

All data were recorded and analyzed using the IBM Statistical Package for Social Sciences (SPSS version 22) Statistics for Windows, Version 22.0 software package. Prior to the primary analysis, the data were screened for completeness, coding errors, and outliers. Prior to the primary analysis, the underlying assumptions were tested to determine whether parametric or non-parametric methods should be employed. The normality of the distribution was assessed using the Kolmogorov–Smirnov test, alongside skewness and kurtosis coefficients. Values of skewness and kurtosis within ±2.0 were considered indicative of an approximately normal distribution. In addition, assumptions relevant to regression-based analyses, including linearity, independence of observations, homoscedasticity, and multicollinearity, were evaluated where appropriate.

Descriptive statistics were expressed as frequencies and percentages for categorical variables and as mean ± standard deviation for continuous variables. The internal consistency reliability of the Health Literacy Scale and Vaccine Refusal Scale was evaluated using Cronbach’s alpha coefficients.

The primary hypothesis of the study was that higher health literacy would be associated with lower vaccine refusal, whereas the secondary hypothesis was that this relationship would vary according to sociodemographic characteristics.

To examine the moderating effects of categorical variables on the impact of health literacy on vaccine refusal, Hayes’ PROCESS macro (Model 1) was utilized [30]. Health literacy was entered as the independent variable, vaccine refusal as the dependent variable, and each sociodemographic characteristic (education level, age, income level, place of residence, and gender) was tested separately as a moderator. Regression coefficients (b), standard errors (SE), 95% confidence intervals (CI), and explained variance (R^2^) values were reported. Statistical significance for all analyses was set at a threshold of *p* < 0.05.

## 3. Results

The results are presented according to the study hypotheses. First, the direct association between health literacy and vaccine refusal was examined. Second, moderation analyses were conducted to determine whether this association differed by educational level, age, income level, place of residence, and gender.

The reliability of the scales used in the study was assessed using Cronbach’s alpha coefficient. The health literacy scale demonstrated acceptable reliability (α = 0.794), while the vaccine refusal scale showed high reliability (α = 0.938), indicating that both scales had sufficient internal consistency for further analyses (Table 1).

Regarding demographic characteristics, 62.71% (*n* = 259) of the participants were aged 18–30 years, 28.57% (*n* = 118) were aged 31–50 years, and 8.72% (*n* = 36) were aged 51–64 years. In terms of gender distribution, 69.25% (*n* = 286) were female, and 30.75% (*n* = 127) were male (Table 2). Educational status indicated that 51.09% (*n* = 211) had an associate degree, 17.68% (*n* = 73) had a bachelor’s degree, 18.16% (*n* = 75) had a postgraduate degree, and 13.08% (*n* = 54) had a high school education or below. Income level was categorized as <22,000 Turkish Lira (TL), 22,000–35,000 TL, 35,001–50,000 TL, 50,001–65,000 TL, and >65,000 TL. With respect to income levels, 37.05% (*n* = 153) earned less than 22,000 TL, 17.43% (*n* = 72) earned between 22,000 and 35,000 TL, 14.04% (*n* = 58) earned between 35,001 and 50,000 TL, 7.99% (*n* = 33) earned between 50,001 and 65,000 TL, and 23.49% (*n* = 97) earned more than 65,000 TL. Additionally, 95.16% (*n* = 393) of participants resided in urban areas, while 4.84% (*n* = 20) lived in rural areas.

The normality of the health literacy and vaccine refusal scores was evaluated using the Kolmogorov–Smirnov test and skewness and kurtosis values. Although some variables showed statistically significant results in the Kolmogorov–Smirnov test (*p* < 0.05), skewness and kurtosis values within ±2.0 indicated no substantial deviation from normality. Therefore, parametric tests were considered appropriate for subsequent analyses (Table 3).

**Figure 1:**
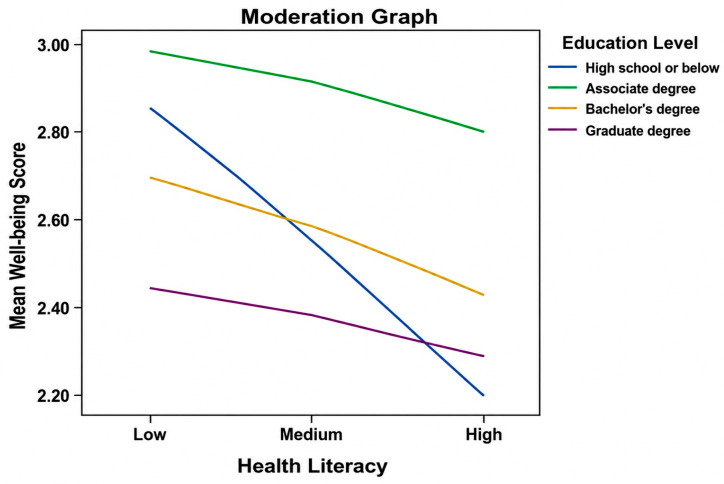
Moderating Effect of Educational Level on the Relationship Between Health Literacy and Vaccine Refusal.

Moderation analysis examining the role of education level revealed that the overall model was statistically significant (F (7405) = 9.58, *p* < 0.001) and explained 14% of the variance in vaccine refusal (R^2^ = 0.14) (Table 4; Figure 1). Health literacy had a significant negative effect on vaccine refusal (b = −0.72, SE = 0.18, t = −3.93, *p* < 0.001), indicating that higher health literacy was associated with lower levels of vaccine refusal. Significant interaction effects were observed for associate degree (b = 0.52, *p* = 0.01) and postgraduate education levels (b = 0.55, *p* = 0.03). These findings suggest that the protective effect of health literacy against vaccine refusal is stronger among individuals with lower educational attainment and weakens as the education level increases. Conditional effects indicated that the negative relationship was strongest in the “high school or below” group (b = −0.72, *p* < 0.001), remained significant but weaker in the associate (b = −0.20, *p* = 0.04) and bachelor’s (b = −0.31, *p* = 0.04) groups, and was not statistically significant in the postgraduate group (b = −0.17, *p* = 0.30).

A moderation analysis was conducted to examine whether age influenced the relationship between health literacy and vaccine refusal (Table 5). The overall model was statistically significant (F (5407) = 6.71, *p* < 0.001) and explained 8% of the variance in vaccine refusal (R^2^ = 0.08). Health literacy was significantly and negatively associated with vaccine refusal (b = −0.30, SE = 0.09, t = −3.34, *p* < 0.001), indicating that individuals with higher levels of health literacy were less likely to exhibit vaccine refusal attitudes. Compared with the reference group (18–30 years), participants aged 31–50 years (b = −0.26, *p* < 0.001) and 51–64 years (b = −0.26, *p* = 0.040) reported significantly lower vaccine refusal scores. However, neither the interaction term between health literacy nor the age category reached statistical significance (31–50 years: b = −0.17, *p* = 0.300; 51–64 years: b = −0.07, *p* = 0.750). These findings indicate that although age was associated with vaccine refusal levels, it did not significantly modify the relationship between health literacy and vaccine refusal. Therefore, the inverse association between health literacy and vaccine refusal appeared to be relatively consistent across age groups.

The analysis of income level as a moderator showed that the overall model was significant [F (9403) = 4.45, *p* < 0.001] and explained approximately 9% of the variance (R^2^ = 0.09) (Table 6). Health literacy significantly reduced vaccine refusal (b = −0.40, *p* < 0.001). Although individuals in the 22,000–35,000 TL income group exhibited higher levels of vaccine refusal compared to the reference group (b = 0.37, *p* = 0.001), the interaction terms were generally not statistically significant, indicating that income level did not meaningfully moderate the relationship.

The moderating role of place of residence (urban vs. rural) was also examined. The model was significant [F (3409) = 9.02, *p* < 0.001] and explained 6% of the variance (R^2^ = 0.06). Health literacy had a significant negative effect on vaccine refusal (b = −0.92, *p* = 0.013). Additionally, residence had a significant main effect, with individuals living in rural areas exhibiting higher levels of vaccine refusal than those living in urban areas (b = 0.47, *p* = 0.008) (Table 7). However, the interaction between health literacy and residence was not statistically significant (*p* = 0.084), indicating no moderating effect. Conditional analyses showed that the effect of health literacy was significant in rural areas (b = −0.32, *p* < 0.001) but not in urban areas (*p* = 0.41).

Finally, the moderating effect of gender was evaluated. The model was statistically significant (F (3409) = 6.68, *p* < 0.001) and explained 4.7% of the variance (R^2^ = 0.05) (Table 8). However, neither the main effect of health literacy (*p* = 0.66) nor the main effect of gender (*p* = 0.67) was statistically significant. Additionally, the interaction term was not significant (*p* = 0.43), indicating that gender did not moderate the relationship between health literacy and vaccine refusal. These findings indicate that gender did not significantly moderate the association between health literacy and vaccine refusal. The relationship between health literacy and vaccine refusal appeared comparable in men and women, suggesting that the influence of health literacy on vaccine-related attitudes does not differ according to gender.

## 4. Discussion

The present study investigated the association between health literacy and vaccine refusal and examined whether this relationship varied according to sociodemographic characteristics. The findings demonstrated a significant inverse association between health literacy and vaccine refusal, indicating that individuals with higher levels of health literacy were less likely to exhibit vaccine-refusing attitudes. More importantly, educational level emerged as a significant moderator of this relationship, suggesting that the protective effect of health literacy against vaccine refusal was strongest among individuals with lower educational attainment and gradually weakened as educational level increased. In contrast, age, gender, income level, and place of residence did not demonstrate consistent moderating effects. These findings highlight the importance of considering educational background when designing health literacy-based interventions aimed at reducing vaccine refusal and improving public acceptance of vaccination programs.

The reliability analysis showed that both measurement tools had acceptable-to-excellent internal consistency. The Cronbach’s alpha value of the Health Literacy Scale was 0.794, indicating acceptable reliability, whereas the Vaccine Refusal Scale demonstrated very high reliability with a Cronbach’s alpha value of 0.938. These findings suggest that the instruments used in the present study were sufficiently reliable for evaluating health literacy and vaccine refusal attitudes. This is important because previous research has emphasized that valid and reliable measurement of health literacy and vaccine-related attitudes is essential for accurately identifying the determinants of vaccine hesitancy and refusal [3,5,13].

The sociodemographic distribution of the participants showed that the sample consisted predominantly of young adults, women, individuals with associate-level education, and urban residents. This profile should be considered when interpreting the findings, as vaccine hesitancy is known to vary across demographic and social groups. Previous studies have reported that age, gender, education, income, and residential context may influence vaccine-related attitudes, although these effects are often inconsistent and context-dependent [31,32]. In the present study, the predominance of urban residents may also partly explain why place of residence did not emerge as a robust moderator, because the number of rural participants was relatively limited.

The normality assessment indicated that although health literacy scores showed statistical significance in the Kolmogorov–Smirnov test, skewness and kurtosis values for both health literacy and vaccine refusal were within acceptable limits. Therefore, the use of parametric analyses was considered appropriate. In large samples, normality tests such as the Kolmogorov–Smirnov test may become overly sensitive to minor deviations from normality; therefore, skewness and kurtosis values are commonly evaluated together with formal tests when deciding whether parametric procedures are suitable [33].

The most important finding of the study was that health literacy was negatively associated with vaccine refusal, and this relationship was significantly moderated by educational level. Specifically, the inverse association between health literacy and vaccine refusal was strongest among participants with lower educational attainment and became weaker as educational level increased. This finding is consistent with previous studies showing that higher health literacy is associated with lower vaccine hesitancy and greater vaccine acceptance [5,6,34]. Lorini et al. [5] reported that health literacy is related to vaccine attitudes, vaccination intention, and vaccine uptake, although the direction and strength of this relationship may vary across populations and vaccine types. Similarly, Biasio emphasized that insufficient health literacy may impair individuals’ ability to understand vaccine-related information and may increase susceptibility to misinformation [6].

The inverse association between health literacy and vaccine refusal observed in the present study is consistent with previous findings from Türkiye. Çetin and Sögüt [21] reported that higher health literacy levels were associated with lower vaccine hesitancy among pregnant women, suggesting that health literacy may facilitate informed vaccination decisions and improve confidence in vaccines. While their study primarily focused on the direct association between health literacy and vaccine hesitancy, our findings extend this evidence by demonstrating that the strength of this relationship varies according to educational level. Specifically, the protective effect of health literacy against vaccine refusal was more pronounced among individuals with lower educational attainment and gradually diminished as educational level increased. The present findings extend this literature by showing that the protective role of health literacy may be particularly important among individuals with lower educational attainment.

The moderating effect of education may be explained by the close but non-identical relationship between education and health literacy. Education provides individuals with general cognitive and informational resources, whereas health literacy reflects the ability to access, understand, evaluate, and use health-related information in decision-making. Therefore, among individuals with lower formal education, health literacy may play a more decisive role in shaping vaccine-related attitudes. In contrast, among individuals with higher education, vaccine refusal may be influenced by additional factors such as trust in institutions, perceived vaccine safety, exposure to conflicting information, ideological beliefs, or personal risk perception. This interpretation is supported by previous studies showing that education is associated with vaccine hesitancy, but that higher education does not always translate into vaccine acceptance in a linear manner [31,34].

The findings of the present study may also be interpreted in light of recent evidence from Türkiye. Palloş and Yüksel Kaçan [20] reported that vaccine hesitancy was present even among academics, a population generally characterized by high educational attainment and access to scientific information. This observation is consistent with our finding that the inverse association between health literacy and vaccine refusal became weaker as educational level increased and was no longer statistically significant among postgraduate participants. Together, these findings suggest that higher educational attainment alone may not be sufficient to prevent vaccine refusal.

While health literacy appears to play a protective role, particularly among individuals with lower educational attainment, vaccine-related attitudes among highly educated individuals may be influenced by additional factors such as trust in health authorities, risk perception, personal beliefs, exposure to conflicting information, and confidence in scientific institutions. Therefore, interventions aimed at reducing vaccine refusal should not rely solely on educational status as a proxy for vaccine acceptance but should also address broader cognitive, social, and informational determinants of vaccination behavior.

This interpretation is further supported by recent findings from Türkiye demonstrating that vaccine hesitancy may persist even among academics, a highly educated population. Such observations suggest that educational attainment alone does not guarantee vaccine acceptance and that factors beyond formal education, including trust, risk perception, and exposure to health information, may contribute to vaccine-related decision-making [35].

These findings are also supported by evidence from Kazakhstan showing that higher levels of vaccination literacy were associated with more favorable perceptions of vaccination among university students. Together with our results, these observations suggest that literacy-related competencies may play an important role in shaping vaccine-related attitudes across different populations and sociocultural settings [36].

Age was significantly associated with vaccine refusal levels, but it did not significantly moderate the relationship between health literacy and vaccine refusal. This suggests that although vaccine refusal may differ across age groups, the protective association of health literacy with vaccine refusal remains relatively similar across ages. Previous research has reported mixed findings regarding age and vaccine hesitancy. Some studies have found higher hesitancy among younger individuals, whereas others have reported that age-related differences depend on vaccine type, perceived disease risk, and sociocultural context [31,32]. The present findings support the view that age alone may not be sufficient to explain how health literacy influences vaccine-related attitudes.

Income level was also examined as a potential moderator. Although certain income categories showed differences in vaccine refusal, the overall pattern did not indicate a consistent or robust moderating role of income. This finding suggests that the relationship between health literacy and vaccine refusal may not substantially differ according to income level in this sample. Previous studies have identified socioeconomic status as one of the social determinants of vaccine hesitancy; however, its effect may operate indirectly through access to healthcare, trust, education, perceived vulnerability, and exposure to misinformation [32,35]. Therefore, income may influence vaccine attitudes, but it may not necessarily alter the effect of health literacy unless broader social and psychological variables are also considered.

Gender did not significantly predict vaccine refusal and did not moderate the relationship between health literacy and vaccine refusal. This indicates that the association between health literacy and vaccine refusal was comparable among men and women. Previous literature has reported inconsistent findings regarding gender differences in vaccine hesitancy. Some studies have observed higher hesitancy among women, particularly in relation to concerns about vaccine safety and side effects, whereas others have found no significant gender effect after accounting for other factors [31,32]. The present findings suggest that gender may not be a central factor in explaining vaccine refusal in this sample.

Overall, the findings indicate that health literacy is an important factor associated with vaccine refusal, but its effect is not uniform across all sociodemographic groups. Among the demographic variables examined, education level emerged as the most meaningful moderator. This suggests that health literacy-based interventions may be particularly useful for individuals with lower educational attainment. However, the modest explanatory power of the models also indicates that vaccine refusal is a multifactorial phenomenon that cannot be explained by health literacy alone. Future interventions should therefore combine health literacy improvement with strategies addressing misinformation, institutional trust, perceived vaccine safety, sociocultural beliefs, and access to reliable health information [13,31,32,35].

## 5. Strengths

The present study has several strengths that should be acknowledged. First, the study included a relatively large sample of adults from different sociodemographic backgrounds, allowing the examination of vaccine-related attitudes across diverse population groups. Second, validated and reliable instruments were used to assess both health literacy and vaccine refusal, increasing the credibility of the findings. Third, this study extends the existing literature by examining not only the direct association between health literacy and vaccine refusal but also the potential moderating effects of multiple sociodemographic characteristics. To our knowledge, few studies conducted in Türkiye have investigated whether the association between health literacy and vaccine refusal varies according to educational level, age, income, gender, and place of residence. Therefore, the present findings provide novel insights that may help inform targeted public health interventions aimed at reducing vaccine refusal.

## 6. Limitations

Several limitations should be acknowledged when interpreting the findings of this study. First, the cross-sectional design precludes any causal inferences regarding the relationship between health literacy and vaccine refusal. Therefore, the observed associations should be interpreted as correlational rather than causal. Second, data were collected through self-report questionnaires administered online, which may be subject to bias, social desirability bias, and inaccuracies related to self-perception. Third, although efforts were made to obtain a diverse sample, the use of online recruitment and snowball sampling may have introduced selection bias and limited the representativeness of the study population. In addition, the sample was predominantly composed of younger individuals, women, and urban residents, which may restrict the generalizability of the findings to other demographic groups. Notably, rural residents constituted only a small proportion of the sample, potentially limiting the statistical power to detect residence-related differences. Furthermore, vaccine refusal is a multifactorial phenomenon influenced by psychological, cultural, political, and informational factors. Variables such as trust in healthcare systems, exposure to misinformation, political attitudes, previous vaccination experiences, and perceived vaccine safety were not assessed in the present study and may have contributed to vaccine-related attitudes. Finally, although educational level emerged as a significant moderator, the moderation models explained a relatively modest proportion of the variance in vaccine refusal, suggesting that additional determinants remain to be explored in future research.

## 7. Conclusions

Higher health literacy was associated with lower levels of vaccine refusal among adults in Türkiye. Among the sociodemographic variables examined, educational level emerged as the only significant moderator, indicating that the inverse association between health literacy and vaccine refusal was strongest among individuals with lower educational attainment and gradually weakened as educational level increased. In contrast, age, gender, income level, and place of residence did not demonstrate robust moderation effects. These findings suggest that health literacy represents an important factor associated with vaccine-related attitudes; however, its influence may vary across educational groups. Public health interventions aimed at reducing vaccine refusal may therefore benefit from incorporating health literacy-enhancing strategies, particularly among populations with lower educational attainment. Future longitudinal and nationally representative studies are warranted to clarify the mechanisms underlying vaccine refusal and to identify additional social, behavioral, and contextual factors that influence vaccination attitudes.

## Figures and Tables

**Table 1:** Reliability Analysis of the Study Scales.

Scale	Cronbach’s Alpha
Health Literacy Scale	0.794
Vaccine Refusal Scale	0.938

**Table 2:** Distribution of Participants According to Sociodemographic Characteristics.

Variable	Category	*n*	%
Age	18–30 years	259	62.71
	31–50 years	118	28.57
	51–64 years	36	8.72
Gender	Male	127	30.75
	Female	286	69.25
Educational Level	High school or below	54	13.08
	Associate degree	211	51.09
	Bachelor’s degree	73	17.68
	Postgraduate degree	75	18.16
Income Level	<22,000 TL	153	37.05
	22,000–35,000 TL	72	17.43
	35,001–50,000 TL	58	14.04
	50,001–65,000 TL	33	7.99
	>65,000 TL	97	23.49
Place of Residence	Urban	393	95.16
	Rural	20	4.84

Abbreviation: TL, Turkish Lira.

**Table 3:** Assessment of Normality for Study Variables.

Variable	Kolmogorov–Smirnov			Skewness	Skewness	Mean ± SD
	Statistic	SD	*p*			
Health Literacy Scale	0.07	413	0.00	0.14	0.12	3.76 ± 0.48
Vaccine Refusal Scale	0.04	413	0.17	0.09	−0.40	2.71 ± 0.70

**Table 4:** Moderating Effect of Educational Level on the Association Between Health Literacy and Vaccine Refusal.

Variable/Term	b	SE	t	*p*	%95 CI
Constant	2.52	0.1	25.05	<0.001	[2.32, 2.72]
Health Literacy (X)	−0.72	0.18	−3.93	<0.001	[−1.08, −0.36]
Education W1 (associate degree)	0.37	0.11	3.38	0.001	[0.16, 0.59]
Education W2 (bachelor’s degree)	0.05	0.13	0.42	0.67	[−0.20, 0.30]
Education W3 (Lisans üzeri)	−0.15	0.13	−1.18	0.24	[−0.40, 0.10]
Int 1 (X × W1)	0.52	0.21	2.49	0.01	[0.11, 0.93]
Int 2 (X × W2)	0.41	0.24	1.71	0.09	[−0.06, 0.87]
Int 3 (X × W3)	0.55	0.25	2.21	0.03	[0.06, 1.03]

Int: Interaction term; SE, standard error; CI, confidence interval.

**Table 5:** Moderating Effect of Age on the Association Between Health Literacy and Vaccine Refusal.

Variable/Term	b	SE	t	*p*	%95 CI
Constant	2.8	0.04	65.63	<0.001	[2.72, 2.89]
Health Literacy (X)	−0.3	0.09	−3.34	<0.001	[−0.47, −0.12]
Age W1 (31–50 years)	−0.26	0.08	−3.34	<0.001	[−0.41, −0.11]
Age W2 (51–64 years)	−0.26	0.13	−2.02	0.04	[−0.50, −0.01]
Int 1 (X × W1)	−0.17	0.16	−1.04	0.3	[−0.49, 0.15]
Int 1 (X × W1)	−0.07	0.24	−0.31	0.75	[−0.54, 0.39]

Int: Interaction term; SE, standard error; CI, confidence interval.

**Table 6:** Moderating Effect of Income Level on the Association Between Health Literacy and Vaccine Refusal.

Variable/Term	b	SE	t	*p*	%95 CI
Constant	2.52	0.1	25.05	<0.001	[2.32, 2.72]
Health Literacy (X)	−0.72	0.18	−3.93	<0.001	[−1.08, −0.36]
Income W1 (22,000–35,000 TL)	0.37	0.11	3.38	0.001	[0.16, 0.59]
Income W2 (35,001–50,000 TL)	0.05	0.13	0.42	0.67	[−0.20, 0.30]
Income W3 (50,001–65,000 TL)	−0.15	0.13	−1.18	0.24	[−0.40, 0.10]
Income W4 (>65,000 TL)	0.52	0.21	2.49	0.013	[0.11, 0.93]
Int 1 (X × W1)	0.41	0.24	1.71	0.088	[−0.06, 0.87]
Int 2 (X × W2)	0.55	0.25	2.21	0.028	[0.06, 1.03]
Int 3 (X × W3)	2.52	0.1	25.05	<0.001	[2.32, 2.72]
Int 4 (X × W4)	−0.72	0.18	−3.93	<0.001	[−1.08, −0.36]

Int: Interaction term; SE, standard error; CI, confidence interval.

**Table 7:** Moderating Effect of Place of Residence on the Association Between Health Literacy and Vaccine Refusal.

Variable/Term	b	SE	t	*p*	%95 CI
Constant	2.22	0.19	11.96	<0.001	[1.86, 2.59]
Health Literacy (X)	−0.92	0.37	−2.49	0.013	[−1.64, −0.19]
Place of Residence (Urban)	0.47	0.18	2.68	0.008	[0.13, 0.82]
Int 1 (X × (Residence)	0.6	0.35	1.73	0.084	[−0.08, 1.28]

Int: Interaction term; SE, standard error; CI, confidence interval.

**Table 8:** Moderating Effect of Gender on the Association Between Health Literacy and Vaccine Refusal.

Variable/Term	b	SE	t	*p*	%95 CI
Constant	2.66	0.13	20.57	0.00	[2.4068, 2.9154]
Health Literacy (X)	−0.11	0.26	−0.43	0.66	[−0.6253, 0.3994]
Gender (W)	0.03	0.07	0.42	0.67	[−0.1138, 0.1761]
Int_1_ (X × Gender)	−0.12	0.15	−0.78	0.43	[−0.4127, 0.1773]

Int: Interaction term; SE, standard error; CI, confidence interval.

## Data Availability

The datasets used and/or analyzed during the current study are available from the corresponding author on reasonable request.

## Supplementary Materials

The following supporting information can be downloaded at: https://www.mdpi.com/article/10.3390/vaccines14070582/s1, File S1: The Moderating Role of Demographic Variables in the Influence of Health Literacy on Vaccine Refusal.
