# Supplementary material for: The Moderating Role of Demographic Variables in the Effect of Health Literacy on Anti-Vaccination Sentiment
Source: Vaccines (Basel). 2026 Jun 30;14(7):582. doi: 10.3390/vaccines14070582 (PMC13417068; doi:10.3390/vaccines14070582)
Supplement: Supplementary file 1 [file vaccines-14-00582-s001.zip › vaccines-4392328-supplementary.pdf]

The Moderating Role of Demographic Variables in the Influence of Health Literacy on Vaccine Refusal

Dear Participants;

This article ***discusses the moderating role of demographic variables in the impact of health literacy on vaccine refusal.*** We invite you to participate in the research entitled [Research Title]. This research is purely for scientific purposes, and the information you provide in the survey will be kept confidential, held only by the researcher, and will not be shared with third parties or institutions. Your participation in this study is entirely voluntary, and it is estimated that you will need to dedicate approximately 10 minutes. Therefore, it is important that you answer the questions in the survey completely and honestly for the reliability and accurate results of the study.

Thank you for your participation.

Lecturer İlkay ALTUNSOY ilkay.altunsoy@atlas.edu.tr

Research Assistant Berkay KARGILI berkay.kargili@yeniyuzyil.edu.tr

Prof. Dr. Abdulhalim ŞENYİĞİT abdulhalim.senyigit@atlas.edu.tr

Consent Statement

I have read and understood the above explanations. I have received sufficient information about the purpose, scope, and participation requirements of the research. I understand that my participation is entirely voluntary and that I can withdraw from the study at any time.

Do you agree to participate in this research?

☐ Yes, I voluntarily agree to participate in the research.

☐ No, I don't want to participate in the research.

Personal Information Form

Age: (1) 18-30 (2) 31-50 (3) 51-64 (4) 65 years and over

Education level: (1) Primary school (2) Secondary school (3) High school (4) Undergraduate (5) Postgraduate

Gender: (1) Female (2) Male

Income level: (1) Low (2) Medium (3) High

Place of residence: (1) City (2) Rural

Anti-Vaccination Scale

This section contains items from the "Vaccine Hesitancy Scale". Please mark the item that best reflects your opinion regarding vaccines.		I absolutely disagree.	I disagree.	I partially agree.	I agree	I absolutely agree.
A1	If everyone is vaccinated, diseases will decrease.					
A2	Vaccination is an effective way to protect health.					
A3	I trust the vaccines recommended by the government.					
A4	Vaccination is the strongest measure against infectious diseases.					
A5	Vaccination is an important safeguard for our health.					
B1	I'm worried about the side effects of vaccines.					
B2	I'm afraid the vaccine might cause autism or learning disabilities.					
B3	Vaccines can cause many diseases.					
B4	Vaccines benefit those who produce them more than they benefit people's health.					
B5	Vaccines have both benefits and drawbacks.					
B6	Vaccines contain toxic substances.					
C1	Traditional methods offer better protection than vaccines.					
C2	I would rather get the disease than get vaccinated to gain immunity.					
C3	If I could, I would abolish mandatory vaccination.					
C4	Vaccination should be optional, not mandatory.					
C5	If I could go back to my childhood, I wouldn't get vaccinated.					
D1	I'm afraid of needles, so I won't get vaccinated.					
D2	I will not get vaccinated due to my religious beliefs.					
D3	I won't vaccinate my child because vaccines can cause permanent illnesses.					
D4	Since the other children have been vaccinated, my child doesn't need to be vaccinated.					

D5	Vaccination is unnecessary because infectious diseases are rare.					

Health Literacy Scale

contains items<br><br>from the "Health Literacy Scale." The following statements are designed to measure how you find, understand, and use health-related information. Please read each statement carefully and indicate how applicable it is to you. There are no right or wrong answers; you are simply expected to reflect your own experiences. **"Health-related information" includes: (doctor's statements, prescriptions, drug leaflets, appointment systems, laboratory results, health brochures, hospital/pharmacy guidelines, e-health application, vaccination campaigns, and similar).**		I absolutely disagree.	I disagree.	I partially agree.	I agree	I absolutely agree.
F1	There is material I haven't been able to read.					
F2	The text is too small for me to read.					
F3	It's quite difficult for me to understand the content.					
F4	Reading the instructions and brochures takes a very long time.					
F5	I need someone's help to read the instructions and brochures.					
I1	I gather information from various sources.					
i2	I get the information I want.					
i3	I try to understand the information I receive.					
i4	I share my thoughts about my illness with the people around me.					
i5	I apply the knowledge I gain in my daily life.					
E1	I consider whether the information is applicable to me.					
E2	I consider whether the information is credible.					
E3	I check whether the information is valid and reliable.					
E4	I gather information to make informed decisions about my health.					
